# Cognitive telerehabilitation in neurological patients: systematic review and meta-analysis

**DOI:** 10.1007/s10072-021-05770-6

**Published:** 2021-11-25

**Authors:** Luisa Cacciante, Camilla della Pietà, Sebastian Rutkowski, Błażej Cieślik, Joanna Szczepańska-Gieracha, Michela Agostini, Pawel Kiper

**Affiliations:** 1 Laboratory of Rehabilitation Technologies, IRCCS San Camillo Hospital, Venice, Italy; 2 Faculty of Physical Education and Physiotherapy, Opole University of Technology, Opole, Poland; 3 Faculty of Health Sciences, Jan Dlugosz University, Czestochowa, Poland; 4 Department of Physiotherapy, University School of Physical Education, Wroclaw, Poland; 5 Department of Neuroscience, Section of Rehabilitation, University-General Hospital of Padova, Padua, Italy; 6 Physical Medicine and Rehabilitation Unit, Azienda ULSS 3 Serenissima, Venice, Italy

**Keywords:** Telerehabilitation, Cognitive treatment, Cognition disorders, Cognitive impairments

## Abstract

Telerehabilitation (TR) seems to be an encouraging solution for the delivery of cognitive treatments in patients with neurological disorders. This study was aimed to analyze and synthesize the evidence on the efficacy of cognitive TR interventions in patients with neurological diseases, compared with conventional face-to-face rehabilitation. From a total of 4485 records, 9 studies met the inclusion criteria for qualitative analysis. At the end of the process, 7 studies remained for quantitative analysis. By comparing TR with face-to-face treatments for cognitive impairments, we assessed improvements in global cognitive domain (Mini Mental State Exam) (MD = −0.86; 95% CI −2.43, 0.72, *I*^2^ = 0%), in learning and memory domains (SMD = 0.26, 95% CI −0.22, 0.74, *I*^2^ = 24%), in verbal fluency (SMD = 0.08, 95% CI −0.47, 0.62, *I*^2^ = 0%), and in executive functions (i.e., problem-solving, central processing speed and working memory) (SMD = 0.38, 95% CI 0.06, 0.71, *I*^2^ = 0%). In all the included studies, improvement in the performance of the TR groups was comparable to that achieved through face-to-face intervention. Significant differences between those two modalities of providing treatments were observed for working memory and total executive function comparison, in favor of TR. The results of this study can sustain the efficacy of TR and its application for the treatment of neurological patients, especially when treated for executive function impairments.

**Supplementary Information:**

The online version contains supplementary material available at 10.1007/s10072-021-05770-6.

## Introduction

Telemedicine is one of the treatment options used to deliver healthcare. The World Health Organization has adopted the following broad description: “The delivery of health care services, where distance is a critical factor, by all health care professionals using information and communication technologies for the exchange of valid information for the diagnosis, treatment and prevention of disease and injuries, research and evaluation, and for the continuing education of health care providers, all in the interests of advancing the health of individuals and their communities” (W. H. O. Group Consultation on Health Telematics [22]). It encompasses a broad range of services: assessment, treatment, monitoring, prevention, intervention, supervision, education, consultation, and counseling, all directed to support individuals with disabilities [4].

This technology has received the greatest interest in the last year, due to the consequences of COVID-19 pandemic for health care services and assistance. Indeed, COVID-19 has had a profound impact on the organization of rehabilitation in all countries [15]. During the lockdown, all kinds of treatment, diagnostics, and counselling experienced significant limitations [6]. Some of the health services were suspended, while others were limited only to emergency situations [9]. In this context, it becomes fundamental to develop new strategies to ensure the continuity of care, and telemedicine seems to be the solution to provide some services at a distance. In particular, in the rehabilitation field, the delivery of rehabilitation services via information and communication technologies is defined as telerehabilitation (TR) [25]. It has been primarily developed to reduce in-patient hospital stay and to facilitate access to services for those patients who have motor disabilities or environmental barriers that make difficult reaching rehabilitation centers after hospital discharge. Furthermore, benefits of TR include the delivery of prolonged therapies tailored to patients’ needs while at the same time making significant savings on costs [1].

Based on videoconference, TR services can be delivered in two ways: synchronous and asynchronous [16]. The first modality is based on two-way videoconferencing with the presence of a therapist, so that patients and therapists work simultaneously, and the therapist delivers rehabilitation treatments in real time. The second approach (asynchronous) does not require the presence of a therapist and allows to provide self-administered computer-based exercises [16]. In this field, there is a growing body of literature that shows potential regarding the application of TR. However, due to methodological and practical concerns, it is difficult to find conclusive evidence on the efficacy of TR compared to conventional face-to-face treatment both for motor recovery [1] and speech and language treatment [24].

Also, for cognitive rehabilitation, there is a need to improve cognitive treatment programs. Cognitive impairments can be found both in patients with brain injuries (traumatic or vascular) and in patients with neurodegenerative pathologies, in which cognition disorders have a progressive course that eventually culminates in global cognitive impairment and compromised functional independence [3]. A critical aspect of cognitive training programs is that the interventions that seemed to be promising have involved intensive in-person sessions that are unlikely to be cost-effective or feasible for large-scale implementation [8].

Therefore, TR seems to be the best solution to face the increasing need for delivery of alternative kinds of cognitive treatments because of the growing social demand and cost of healthcare. Indeed, in the current pandemic situation, the relevance of TR, which could reduce unnecessary hospitalization, seems to be particularly important. Nevertheless, previous studies showed that several barriers and limitations, such as administrative licensing, medicolegal ambiguity, financial sustainability, and the lack of technological infrastructures, still remain and limit the spreading of TR [2, 21].

Thus, given the absence of systematic reviews and meta-analyses related to TR treatment for cognitive impairment among patients with neurological diseases (i.e., stroke, traumatic brain injury, Parkinson’s disease, multiple sclerosis, mild cognitive impairment), the aim of this systematic review was to analyze and synthesize the evidence on the efficacy of cognitive TR interventions in patients with neurological diseases, compared with conventional face-to-face rehabilitation.

## Methods

The study design was set as a systematic review and meta-analysis and was conducted according to the PRISMA guidelines [17]. The protocol was registered a priori in the PROSPERO database under the following registration number CRD42019137721.

### Electronic searches

Publications were searched in PubMed, Embase, Web of Sciences, Scopus, and the Cochrane Library. The last search was launched on the 30th of April 2020. A detailed description of the search strategy is presented in the supplementary materials (Appendix A).

### Study selection

In this review, we included (1) publications designed as a randomized controlled trial (RCT), with (2) participants being adults with a neurological disease (e.g., dementia, Parkinson’s disease, multiple sclerosis, stroke, traumatic brain injury, cognitive impairment), (3) an intervention defined as a TR (either synchronous or asynchronous), and (4) at least one of the outcomes that assessed the cognitive status. The review included only publications in English. Gray literature was not searched in this review. For study selection through abstract screening, six reviewers were divided into three groups (two reviewers for each group). Abstracts that had to be screened were divided equally into the three groups. The reviewers, independently, screened studies that were identified through the electronic search engines already mentioned, based on title and abstract, using an inclusion/exclusion criteria template. A third reviewer was selected from each of the three groups to solve any disagreements in one of the groups. At the end of this process, full text of the articles were obtained, and the same procedures were used for full text screening and for the assessment of the methodological quality (risk of bias assessment).

### Outcomes

The main outcome of this systematic review was to analyze the improvement in cognitive domains in patients who underwent TR versus conventional face-to-face treatment. We assessed improvements in global cognitive domain, through the analysis of the results from the Mini Mental State Exam (MMSE), in learning and memory domains, in language abilities through analysis of performances in verbal fluency, and in executive functions, through the analysis of different mental skills (i.e., problem-solving, central processing speed, and working memory). Secondary outcomes were related to quality of life, patient satisfaction, feasibility, and cost-effectiveness of TR assessed with questionnaires related to quality of life, patient satisfaction, and feasibility of TR. Also, financial reports were considered to assess the cost-effectiveness of the system.

### Data extraction and management

A data extraction form was filled with all the relevant data, i.e., authors and year of publication, study design, participants’ characteristics, attrition from intervention, co-interventions, number of participants, age, details of intervention in accordance with the Template for Intervention Description and Replication (TIDieR) checklist [11], outcome measures, and when they were administered.

### Assessment of risk of bias in included studies

Studies included in the review underwent a methodological quality assessment for risk of bias using the Cochrane Risk of Bias Tool [10]. We evaluated the following domains: (1) selection bias—sequence generation, allocation concealment; (2) detection bias: blinding of outcome assessment; (3) attrition bias: incomplete outcome data; and (4) reporting bias: selective reporting. We decided to omit the domain that assesses the blinding of participants, as blinding is not possible in most cases and because we deemed that this domain is related to the nature of the intervention rather than to study quality, as Laver et al. already stated [13]. We coded risk of bias for each domain as “high risk,” in case of a high possibility in the occurrence of bias; “low risk,” in case of a low possibility of bias; “unclear risk,” when we could not exactly define the real incidence of bias. Detailed results of the risk of bias assessment are included in the supplementary materials (Appendix B).

### Measures of treatment effect

We used Review Manager 5.3 (RevMan 2014) to conduct review, to record descriptive information for each study in the characteristics of the included studies tables, to assess the methodological quality of trials through the risk of bias tables, and for statistical analysis. Treatment effects were evaluated using mean difference (MD) for homogeneous outcome measures or standardized mean difference (SMD) for the outcomes evaluated with different scales. Confidence interval (CI) for continuous outcomes was identified at 95%.

### Assessment of heterogeneity

Statistical heterogeneity was assessed with the *I*^2^ statistic, establishing the cutoff value at 50% and considering intervention and outcome measures.

### Data synthesis

We conducted a meta-analysis based on random-effects model or fixed model with 95% CI using RevMan 5.3. We explored heterogeneity as detailed above.

### Subgroup analysis

We planned a subgroup analysis based on different skills within a single domain (e.g., problem-solving, central processing speed, and working memory, within executive functions).

## Results

### Results of the search

**Figure 1:**
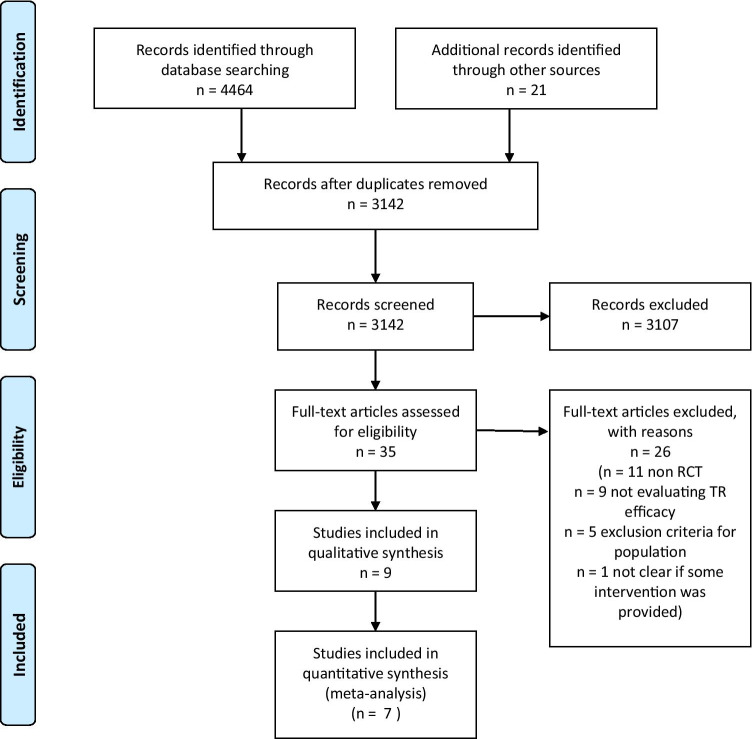
Flow diagram of the studies

Our search identified 4464 results from 5 electronic databases. Moreover, we found 21 additional records from hand search, resulting in 4485 records, overall. After removing 1343 duplicates, 3142 abstracts remained for screening. We excluded 3107 records with unrelated target topics and then assessed for eligibility a total of 35 full text articles. After full-text screening, 9 studies met the inclusion criteria for qualitative analysis. At the end of the process, 7 studies remained for quantitative analysis. The PRISMA flowchart of the review process is shown in Figure 1.

### Included studies

All the included studies were RCTs focusing on the use of TR for cognitive impairments. Among the included studies, one [5] included participants with mild cognitive impairment subjective cognitive impairment or with Alzheimer’s disease. Two studies [7, 20] focused on the treatment of patients with multiple sclerosis (MS), whereas Jelcic et al. included only participants with AD [12]. One study [14] considered TR for cognitive impairments resulting from acquired brain injury and three trials [16, 23, 26] included post-stroke patients. Finally, Poon et al. [18] treated participants with mild dementia or mild cognitive impairment. The overall number of participants included within trials was 505, with 227 patients involved in TR programs and 278 patients treated in control groups. Synchronous TR was provided in the following studies: Burton et al., Poon et al., Jelcic et al., Meltzer et al., Zhou et al., and Man et al., whereas Charvet et al., Sandroff et al., and Torrisi et al. used asynchronous TR. All studies analyzed patients with cognitive impairments, except studies by Meltzer et al. and Zhou et al., who enrolled patients with both cognitive and language disorders. Indeed, study by Meltzer et al. included participants with cognitive-linguistic communication disorders and stratified them to an aphasic or a cognitive group [16]. Treatments for both aphasic and cognitive groups were administered both in-person and remotely. Study by Zhou et al. included patients with language and cognitive impairments and delivered both language and cognitive training to all participants [26].

In relation to the study’s aim, Burton et al. aimed at comparing goal-oriented face-to-face cognitive rehabilitation with videoconferencing, in order to determine whether TR is feasible. The authors cautiously suggest that cognitive rehabilitation can be adapted to telehealth videoconferencing for older adults with subjective and objective memory impairment. TR approach appeared feasible but still requires dependence on caregivers and therapists for manipulating materials; thus, some modifications are needed [5].

Charvet et al. evaluated the benefit of an asynchronous TR program compared with ordinary computer games in adults with MS. They found significant differences in the main outcome of cognitive functioning assessed with a neuropsychological composite score, consisting of a battery of neuropsychological tests (i.e., Paced Auditory Serial Addition Test, WAIS-IV Letter Number Sequencing and Digit Span, Selective Reminding Test, Brief Visuospatial Memory Test-Revised, and Delis-Kaplan Executive Function System Trails). Participants in the TR group showed greater improvements (*P*=0.03). The authors stated that this TR approach can allow for rapid recruitment and high compliance. Furthermore, it can be applied to other neurological conditions associated with cognitive dysfunction [7].

Jelcic et al. aimed to compare the effects of a lexical-semantic stimulation training for patients with AD, delivered via videoconference or face-to-face. Results showed a significant improvement in global cognitive performance assessed with the MMSE for both TR (*P*=0.03) and face-to-face (*P*=0.01) treatments compared to baseline values. In depth, the between-group comparison showed no differences between the groups in visual-spatial memory (measured by means of ROCF Delayed Recall) and in visual-spatial abilities (scored by means of ROCF Copy). Nevertheless, attention abilities assessed with the Digit Cancellation Test improved significantly only in the TR group (*P*=0.01). The authors concluded that TR technology for cognitive rehabilitation was reported as a valuable and well-accepted technology by the patients [12].

In their study, Man et al. wanted to evaluate the effectiveness of cognitive TR in the context of problem-solving for persons with ABI. In this case, the authors stated that the statistically significant improvement in problem-solving skills in the TR group suggests the effectiveness of this approach for improving cognitive functions in patients with ABI. This approach can therefore yield results comparable to face-to-face training [14].

Similarly, the aim of the study by Poon et al. was to examine and compare the feasibility, acceptability, and clinical outcome of a cognitive intervention program for patients with mild cognitive impairment and mild dementia using TR versus a conventional face-to-face treatment. Participants in both face-to-face and TR groups achieved significant improvements in cognitive functions (*P*<0.001) between pre- and post-training, but no significant differences were found between groups. The authors concluded that TR was a feasible and an acceptable method to provide cognitive assessments and treatments to persons with mild cognitive deficit [18].

Sandroff et al. examined the efficacy of an Internet-delivered physical training intervention for improving cognitive processing speed, measured with the Symbol Digit Modalities Test and walking performance, assessed with the 6-minute walk test, in patients with MS. The authors reported that cognitive processing speed scores increased in the intervention condition for those with mild disability (*d* = 0.41), whereas there was minimal change for those with moderate disability (*d* = −0.12); those in the control condition had minimal change regardless of their disability status (*d* = 0.10). By comparison, the intervention increased walking performance (*d* = 0.08) regardless of the disability status, whereas walking performance decreased in the control condition (*d* = −0.06). The authors recommended the use of Internet-delivered physical training intervention on cognitive and walking performance in this population [20].

In the study by Meltzer et al., the authors evaluated the effectiveness of TR by conducting a randomized non-inferiority trial. For the cognitive domain, the 11 participants diagnosed with cognitive-linguistic communication disorders post-stroke exhibited significant improvements between pre- and post-test in memory and language functions. However, no significant differences between TR and in-person group were observed for all cognitive domains assessed (i.e., language, memory, executive functions, attention, and visuo-spatial abilities) [16].

Torrisi et al. evaluated the effectiveness of TR for cognitive impairments in post-stroke patients. Results showed significant differences between the TR group and control group for phonemic fluency (*P* = 0.04) and for the Rey Auditory Verbal Learning Test I (*P* = 0.03). The authors concluded that TR for cognitive disorders following stroke is effective, and patients perceived constant attention to them, maintaining a high level of motivation [23].

Finally, Zhou et al. investigated the efficacy of a computerized training for aphasia that combined speech-language and cognitive training delivered on an in-patient unit or via TR to discharged patients. They assessed language function with the Western Aphasia Battery and practical communication skills with the Communicative Abilities in Daily Living Test at two time points (T1 and T2). Results demonstrated a significant effect of time (*P* < 0.001) but not of group (*P* > 0.75). The authors concluded that this combined form of computerized training promoted aphasia recovery more effectively than a traditional training, for both hospitalized and discharged patients [26].

More detailed information regarding the characteristics of the included studies is presented in Table 1.

**Table 1:** Characteristics of included studies

Burton & O'Connell [5]	
Methods	RCT
Participants	6 participants with subjective cognitive impairment (*n* = 4), MCI (*n* = 1), or dementia due to Alzheimer disease (*n* = 1) randomly allocated to telehealth videoconferencing (*n* = 3) or in-person cognitive rehabilitation (*n* = 3)
Intervention	1. Telehealth videoconferencing Intervention: Individually tailored cognitive rehabilitation via videoconference. Materials and procedures: all participants participated in an in-person assessment. Following the assessment, goals for cognitive rehabilitation were set collaboratively, and baseline performance and satisfaction were measured. Measurement occurred through telehealth. Following 3 weeks of baseline measurement, each participant’s first goal was addressed in the subsequent cognitive rehabilitation sessions. A new goal, or set of goals, was introduced every 3 weeks. Provided by: a senior doctoral student in clinical psychology and supervised by a neuropsychologist Delivery: via videoconference Regimen: 1-h session, once a week, for 8 weeks 2. In-person treatment Intervention: Individually tailored in-person cognitive rehabilitation. Materials and procedures: all participants participated in an in-person assessment. Following the assessment, goals for cognitive rehabilitation were set collaboratively, and baseline performance and satisfaction were measured. Measurement occurred in-person. Following 3 weeks of baseline measurement, each participant’s first goal was addressed in the subsequent cognitive rehabilitation sessions. A new goal, or set of goals, was introduced every 3 weeks. Provided by: a senior doctoral student in clinical psychology and supervised by a neuropsychologist Delivery: via videoconference Regimen: 1-h session, once a week, for 8 weeks
Outcome measures	Two sets of measures were used in this study: pre-post measures and weekly measures. Three baseline measures (B1, B2, B3) and 8 weeks of cognitive rehabilitation (CR1-CR8). Battery: Rivermead Behavioral Memory Test III (RBMT-III), Delis Kaplan Executive Function System (D-KEFS), Verbal Fluency Subtest, Test of Everyday Attention (TEA), Quality of Life in Alzheimer Disease (QoL-AD), World Health Organization Quality of Life Assessment, Short Version (WHOQOL-BREF), Zarit Burden Inventory (ZBI)
Notes	
Charvet et al. [7]	
Methods	RCT
Participants	135 participants with multiple sclerosis divided into two groups: ACR (*n* = 74) vs. active control (*n* = 61)
Intervention	1. Adaptive Cognitive Remediation (ACR) Intervention: ACR is an online adaptive cognitive training program with a set of 15 exercises targeting speed, attention, working memory, and executive function through the visual and auditory domains. Each exercise employed multiple stimulus sets designed to span relevant dimensions of real-world stimuli. The goal of the training exercises is to improve the speed and accuracy of brain information processing while engaging neuromodulatory systems, and allow the generalization of training to improvement cognitive performance in real-world situations. Materials and procedures: Participants were instructed to train in their assigned condition. All participants used a study-provided laptop computer, peripheral equipment including headphones, and a user guide with directions for the use of their assigned program. They had ongoing access to technical support as well as a scheduled weekly check-in phone call. Provided by: a study technician conducted the weekly check-in phone calls Delivery: computer-based Regimen: 1 h per day, 5 days per week, over 12 weeks (targeting 60 h of total program use). 2. Active control condition Intervention: The active control condition was a software gaming suite. These games served as an active placebo control, designed to account for nonspecific treatment effects including interactions with research personnel, and computer-based game-playing. Materials and procedures: Participants were provided a set gaming schedule and were instructed to play games in an arrangement that mirrored to the active condition. The games were selected for “face validity” as having cognitive benefit (e.g., word puzzles) but did not include the active condition's program design features to drive learning or maintain user challenge. All participants used a study-provided laptop computer, peripheral equipment including headphones, and a user guide with directions for the use of their assigned program. They had ongoing access to technical support as well as a scheduled weekly check-in phone call. Provided by: a study technician conducted the weekly check-in phone calls Delivery: computer-based Regimen: 1 h per day, 5 days per week, over 12 weeks (targeting 60 h of total program use).
Outcome measures	A battery of neuropsychological tests was administered at baseline and study end visits. Paced Auditory Serial Addition Test (PASAT), WAIS-IV Letter Number Sequence, WAIS-IV Digit Span Backwards, Selective Reminding Test, Brief Visuospatial Memory Test-Revised (BVMT-R), Delis-Kaplan Executive Function System Trails
Notes	
Man et al. (2) [14]	
Methods	RCT
Participants	109 patients with acquired brain injury, randomly assigned to one of four groups: computer-assisted training (CCRG) (*n* =30), therapist-administered training (TCRG) (*n* = 30), online interactive computer-assisted training (OCRG) (*n* = 29), and control group (CG) (*n* = 20)
Interventions	1. Computer-assisted training (CCRG) Intervention: computer-assisted, skill-training programme in solving problems using analogies. The self-paced computer-assisted training strategy was complemented with face-to-face support from a therapist if needed. For example, the trainees could clarify queries and request performance feedback from the therapist while in need. The subjects were required to perform regular problem-solving exercises in order to become habitualized in daily problem-solving skills. Materials and procedures: This programme was equipped with interactive multimedia presentations on the knowledge and concepts required for persons with ABI to function independently in daily life. Knowledge or lessons were presented in a linear format (one idea after another), supplemented by video and graphical presentations. Lessons were graded by the level of difficulty above the baseline presentation, allowed the trainees to have more control over the presentation and provided role-playing, positive feedback, and errorless learning strategies. Provided by: Therapists Delivery: computer-based, face-to-face Regimen: 20-session training (each lasted for 45 minutes) in 2 months 2. Online interactive computer-assisted training (OCRG) Intervention: The online programme mirrored the structure and content of the computer-assisted version. Materials and procedures: The treatment programme was developed by using the sharing features of Microsoft’s Net-Meeting software, which reflected the visual layout of the computer screen on the therapist’s side to a remote computer on the patient’s side. The therapist was in full command of the programme, exchanging images and audio through the broadband network to the computer on the subject’s side. High-end video-conference units were employed to achieve appealing visual and audio effects. Similar to the therapist-administered programme, the remote therapist could also demonstrate the analogical problem-solving strategy and using positive feedback and errorless learning strategies in the training. Provided by: Therapists Delivery: via videoconference Regimen: 20-session training (each lasted for 45 minutes) in 2 months 3. Therapist-administered training (TCRG) Intervention: conventional face-to-face, activity-based, cognitive rehabilitation programs, the contents of which were identical to those of the OCRG and CCRG groups. The subjects were required to perform regular problem-solving exercises in order to become habitualized in daily problem-solving skills. Materials and procedures: The TCRG provided the most intensive “human touch” in the training through adopting a similar analogical problem-solving strategy demonstration, positive feedback, and errorless learning strategies as the OCRG and CCRG. According to the respective hierarchy of the problem solving (e.g., basic to function), they were given 10 analogous sources (with solutions and strategies) and target (the trainees provide solutions according to their understanding of the respective source question) problems. The TCRG performed and submitted their homework in a pencil-and-paper answer sheet format. The trainers gave the subject’s feedback on their performance as a consolidation of their problem-solving skills learning as well. Provided by: Therapists Delivery: face-to-face Regimen: 20-session training (each lasted for 45 minutes) in 2 months 4. Control group (CG) Wait-listed group. Participants in CG did not receive any intervention in problem-solving skills during the 2-month study period.
Outcomes	Problem-solving skills and self-efficacy were assessed.
Poon et al. [18]	
Methods	RCT
Participants	22 community-dwelling older subjects with mild dementia or mild cognitive impairments randomized either in a videoconference group (*n* = 11) and a face-to-face (FTF) group (*n* = 11)
Interventions	1. Videoconference Intervention: A total of 12 sessions of assessment and cognitive intervention (CI) were conducted via videoconferencing Materials and procedures: VC units were installed at a social center and Shatin Hospital where the research team was based. The VC systems was linked via broadband (1.5 Megabytes per second bandwidth). A high-resolution document camera was used to project images during assessment and intervention. Provided by: A social worker at the social center was assigned to coordinate the CI program. Delivery: via videoconferencing Regimen: A total of 12 CI sessions were conducted over 6 weeks. 2. Face-to-face Intervention: A total of 12 sessions of assessment and cognitive intervention (CI) were conducted by the face-to-face method Materials and procedures: sessions of assessment and CI conducted face-to-face Provided by: A social worker at the social center was assigned to coordinate the CI program. Delivery: Face-to-face Regimen: A total of 12 CI sessions were conducted over 6 weeks
Outcome measures	Outcome measures: Cantonese version of Mini-Mental State Examination (C-MMSE); Cantonese version of Rivermead Behavioural Memory test (C-RBMT); Hierarchic Dementia Scale (HDS); user satisfaction questionnaire towards VC was distributed to participants and staff.
Sandroff et al. [20]	
Methods	RCT
Participants	82 patients with multiple sclerosis (MS) randomly allocated into physical activity behavioral intervention (*n* = 41) or wait-list control conditions (*n* = 41).
Interventions	1. Intervention condition Intervention: Participants in the intervention condition received a theory-based program for increasing physical activity behavior that was delivered via the Internet, and one-on-one video chat sessions with a behavior-change coach. Materials and procedures: For the physical activity intervention, patients visited a study website, wore a Yamax SW-401 Digiwalker pedometer, completed a log book and used Goal Tracker software, and participated in one-on-one video coaching sessions. The website provided content based on social cognitive theory (SCT) for increasing ambulatory physical activity. The behavioral intervention further involved weekly, one-on-one behavioral coaching sessions via Skype. The sessions were semi-scripted and based on principles of supportive accountability (i.e., encouraging participants to wear the pedometer daily and monitor behavioral change and goal attainment throughout the 6-month intervention). The coaching sessions each consisted of a review of goal setting and progress toward goal attainment, as well as a discussion of strategies and facilitators of behavioral change based on SCT and current website content. Provided by: laboratory personnel Delivery: via the Internet Regimen: 6-month intervention with decreased frequency 2. Wait-list control condition Intervention: Participants in this condition completed the study measures before and after the 6-month period, and then received the intervention as described above once the study reached completion.
Outcome measures	Outcome measures: Symbol Digit Modalities Test (SDMT); 6-minute walk (6MW) test; the abbreviated International Physical Activity Questionnaire (IPAQ), The patient-determined disease steps (PDDS) scale.
Jelcic et al. [12]	
Methods	Pilot study
Participants	Total of 38 participants. 27 participants met the selection criteria and entered the study. They were randomly assigned to three treatment groups: seven patients received lexical-semantic stimulation (LSS) with a teleconference technology (LSS-tele); ten were treated with a face-to-face direct administration of LSS (LSS-direct), and ten control subjects underwent unstructured cognitive stimulation (UCS).
Interventions	1. Lexical-semantic stimulation–teleconference technology (LSS-tele) Intervention: The LSS protocol contained lexical tasks aimed at enhancing semantic verbal processing. The exercises focused on the interpretation of written words, sentences, and stories and were divided into eight main parts: semantic categories, syntagmatic and paradigmatic relationship, level of semantic affinity between words, adequacy of adjectives to the context of the text, part-whole relationship, recognition of nonsense sentences, identification of semantic definition, and context of a short story. In the LSS-tele treatment, the same LSS exercises were delivered through remote control based on telecommunication technology. Materials and procedures: In the LSS-tele protocol, the therapist was based at the Hospital and was connected to a group of patients placed in two elderly day care centers. One trained operator was based in the patients’ room with the aim to guarantee the correct access to the technologies and to facilitate the interaction with the treatment therapist when required. The rehabilitation protocol was provided at distance by a customized system, based on two applications run on two personal computer workstations. The therapist’s interface allowed for control of all the experimental information. The patients’ side of the interface was designed with two windows: one showing the therapist by videoconference, the other displaying the target exercise. Provided by: a neuropsychologist and a trained operator Delivery: via videoconference Regimen: two weekly sessions, lasting 1 h each in the morning, over a period of 3 months 2. Lexical-semantic stimulation–direct (LSS-direct) Intervention: Participants of the LSS-direct group received the LSS intervention by the same face-to-face modality, in the presence of the therapist during the entire session. Materials and procedures: Participants of the LSS-direct group received the LSS intervention by the same face-to-face modality, in the presence of the therapist during the entire session. Provided by: a neuropsychologist Delivery: face-to-face Regimen: two weekly sessions, lasting 1 h each in the morning, over a period of 3 months 3. Unstructured cognitive treatment (UCS) Intervention: Participants of the UCS group were engaged in face-to-face training. Materials and procedures: Exercises consisted of creative work such as practicing manual skills, stimulating fantasy and creativeness, reading the newspaper with active participation and discussion, and improving verbal communication. Provided by: a neuropsychologist Delivery: face-to-face Regimen: two weekly sessions, lasting 1 h each in the morning, over a period of 3 months
Outcome measures	Extensive neuropsychological assessment addressing multiple cognitive domains was given to each subject at study entry and postintervention after 3 months of treatments. Primary outcome measures were (a) global cognitive performance, assessed with the Mini-Mental State Examination (MMSE); (b) lexical-semantic abilities, assessed with the Verbal Naming Test and phonemic and semantic fluency; and (c) semantically related and unrelated immediate and delayed episodic verbal memory, assessed respectively with Brief Story Recall and Rey Auditory Verbal Learning (RAVL) tests. Secondary outcome measures were (a) working memory, assessed with the Forward Digit Span Test; (b) visual-spatial memory, assessed with the Rey–Osterrieth Complex Figure (ROCF) Delayed Recall Test; (c) attention and executive functions, assessed with Digit Cancellation Test and Trail Making Test (A and B); (d) visual-spatial abilities, evaluated with the ROCF Copy Test.
Meltzer et al. [16]	
Methods	Randomized non-inferiority trial
Participants	Participants were randomly assigned to in-person (IP) or telerehabilitation (TR) group: IP Group: 22 participants—16 aphasic (*M* = 62.9 years, *SD* = 11.6); 6 with CLCD (*M* = 63.2 years, *SD* = 8.4) TR Group: 22 participants—17 aphasic (*M* = 66.8 years, *SD* = 11.2); 5 with CLCD (*M* = 60.8 years, *SD* = 10.4)
Interventions	In-person treatment Intervention: tablet-based homework exercises and realistic, customized treatment plans tailored to the needs of each individual client. Materials and Procedures: the study consisted of an in-person assessment before and after a 10-week treatment, with a heavy emphasis on homework exercises completed on a tablet, with weekly therapist contact conducted in-person. Communication partner received training and participated in the weekly contact sessions. The study was not limited to aphasia, but also included clients with cognitive-linguistic communication disorders (CLCD). The therapist conducted a 1-h/week treatment session; in three sessions (weeks 3, 6, and 9), 30 min of each session was devoted exclusively to the communication partner, giving training on Supported Conversation techniques and helping the partner keep the client on track with the treatment program. Provided by: speech and language therapist. Delivery: face-to-face Regimen: 1-h/week treatment for 10 weeks. 2. Telerehabilitation Intervention: tablet-based homework exercises and realistic, customized treatment plans tailored to the needs of each individual client. Materials and procedures: remote therapy sessions were conducted via teleconferencing equipment and software. Participants consulted the therapist using WebEx, a commercial teleconferencing program, except for one participant who preferred to use VSee as they were already familiar with it. Others visited a local site of MBTelehealth, a province-wide network for the provision of health-care services through videoconferencing technology. A few participants went to the therapy site itself for TR treatment, without contact with the treating therapist. The treating therapist conducted 1-h weekly treatment session; in three sessions (weeks 3, 6, and 9), 30 min of the session was devoted exclusively to the communication partner, giving training on Supported Conversation techniques and helping the partner keep the client on track with the treatment program. In some cases, a brief telephone call was conducted between therapy sessions to provide support and to monitor progress, particularly when there were concerns about homework compliance. For homework exercises, the majority of the clients used the commercial software program by TalkPath, which comprises graded exercises in speaking, listening, reading, writing, and paralinguistic cognitive skills including memory. Provided by: speech and language therapist. Delivery: remotely, via teleconferencing equipment and software. Regimen: 1h a week, for 10 weeks.
Outcome measures	Primary outcomes: Western Aphasia Battery-Revised, Part 1 (WAB-R) for people with aphasia; Cognitive-Linguistic Quick Test (CLQT) for participants with Cognitive-Linguistic Communication Disorder (CLCD); Communication Confidence Rating Scale for Aphasia to assess subjective communication confidence in the participants themselves; Communication Effectiveness Index to evaluate the functional competence of participants from a subjective but external perspective. The assessment took place during the first and the last week of intervention and was carried out by a SLP not involved in the treatment administration.
Torrisi et al. [23]	
Methods	Randomized controlled trial
Participants	Forty patients (mean ± SD: age = 55.17 ± 18.37 years; 26% male) affected by cognitive disorders due to either ischemic or hemorrhagic stroke were enrolled and randomized into the control (*n* = 20) or the experimental (*n* = 20) groups, in order of recruitment.
Interventions	1. Telerehabilitation Intervention: The telerehabilitation device VRRS allows the monitoring of patient remotely in his/her home by a real-time interaction, comparable to a vis-a-vis interaction. Materials and procedures: The pictures were presented on a computer screen using customized software. The software allows a remote communication between therapist and patient using an embedded communication platform. In this study, the cognitive module with 3D scenarios was mainly used during the hospital training, whilst 2D exercises were used at home. The exercises performed by the patients included attention, memory, visuo-spatial, and reasoning tasks. The cognitive rehabilitation method chosen was the restorative method (consisting in enhancement of compromised abilities) rather than the compensatory (based on the development of alternative strategies). Provided by: Twice a week, a psychologist monitored the progress of rehabilitation at home through a videoconference. Delivery: Communication with participant based at home through internet connection. Regimen: The EG and the CG performed the same amount of treatment, i.e., five sessions a week, each session lasting about 50 min. 2. Face-to-face treatment Intervention: patients were trained with the same exercises as in telerehabilitation group, but using paper–pencil tools. Materials and procedures: Participants performed a neuropsychological assessment before entering in treatment. Evaluation at baseline (T0), after twelve weeks (T1), and at the end of the protocol, that is 12 weeks later (T2). During the first phase (T0–T1), the two groups underwent different rehabilitative training at our center: the EG patients underwent a cognitive rehabilitation training performed using the VRRS-Evo, whereas the CG patients were trained with the same exercises, but using paper–pencil tools. In the second phase (T1-T2), all the patients were discharged, and the EG continued cognitive rehabilitation using the VRRS Home Tablet including the same exercises carried out in inpatient regimen (three sessions a week, each session lasting about 50 min). Provided by: Twice a week, a psychologist monitored the progress of rehabilitation at home through a videoconference. Delivery: Face-to-face using paper–pencil tools Regimen: The EG and the CG performed the same amount of treatment, i.e., five sessions a week, each session lasting about 50 min.
Outcome measures	Outcomes recorded at baseline (T0), postintervention after 12 weeks (T1), and follow-up after 24 weeks (T2). The neuropsychological battery: (1) Montreal Overall Cognitive Assessment (MOCA); (2) Frontal Assessment Battery (FAB) and Weigl Test; (3) Attentive Matrices (AM) and Trail Making Test (TMT A, B and B-A); (4) Rey Auditory Verbal Learning Test (RAVLT; immediate and differite) and Digit Span; (5) phonemic and semantic verbal fluency; and (6) Hamilton Rating Scale for Anxiety (HRS-D) and Depression (HRS-D).
Zhou et al. [26]	
Methods	RCT
Participants	Forty patients participated in the experiment. Patients were randomly assigned to each group as follows: Group 1: 10 participants, inpatient control group (ICG) Group 2: 10 participants, inpatient cognitive training group (ITG) Group 3: 10 participants, discharge control group (DCG) Group 4: 10 participants, discharge cognitive training group (DTG)
Interventions	1. In-person training Intervention: computerized intervention for aphasia that combined speech-language and cognitive training delivered on an inpatient unit. Materials and procedures: participants were randomly assigned to the combined speech-language and cognitive training group (ITG) or the control group (ICG). The ICG was provided with routine treatment, while the ITG group received computerized speech-language and cognitive training. Provided by: speech and language therapists. Delivery: one-to-one, and face-to-face. Regimen: twice a day, for 14 days. 2.Telerehabilitation Intervention: remote communication training for discharged control group (DCG), with additional computerized speech-language and cognitive training for discharged cognitive training group (DTG). Materials and procedures: for the DTG, remote communication training was adopted with additional communication speech-language and cognitive training. The telerehabilitation training program was adopted from the Wispirit Inc. (66nao.com). The training program included both a speech-language module and a cognitive training module. The training assignment was based on individual’s deficit profile. Training program included a speech-language module and a cognitive module. To enable adaptive training, each task was designed with different levels of difficulty by adjusting the number of stimuli, the size of the stimulus, and the timing of the presentation. Provided by: speech and language therapist. Delivery: one-to-one, via telerehabilitation. Regimen: The DCG group engaged in family topics communication for 30 min per session, 2 times a day for 30 days, and the DTG group engaged in family topics communication for 30 min a day, with additionally computerized speech-language and cognitive training for 30 min a day for 30 consecutive days.
Outcome measures	Western Aphasia Battery (WAB); Communicative Abilities in Daily Living (CADL). Data collection: T1 for baseline and T2 for end of treatment (after 14 days for inpatient groups and after 30 days for discharged patients)

### Excluded studies

After full-text screening, we excluded a total of 26 studies. Eleven studies were considered ineligible as non-RCTs, whereas other 9 studies did not evaluate the effect of TR on cognitive impairments (i.e., they evaluated the effects of computer-based treatment). One study was excluded because it was not clear if participants carried out cognitive training and, if so, what kind of treatment the therapists provided. Finally, further 5 studies were excluded because the studied population did not meet the inclusion criteria for this review.

### Risk of bias in the included studies

**Figure 2:**
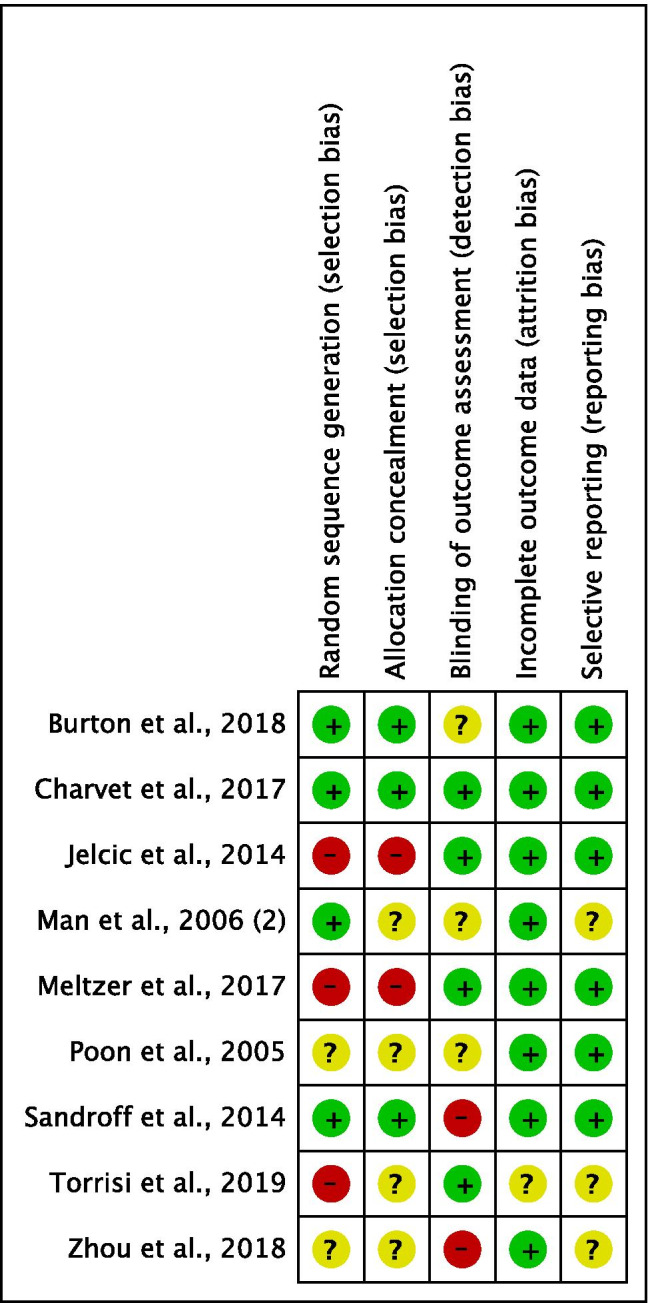
Risk of bias summary

Figure 2 shows the risk of bias in the included studies.

- Random sequence generation (selection bias): Four studies were assessed with a low risk of bias, as the authors described a random component in the sequence-generation process, whereas three studies were judged with a high risk of bias, as randomization procedures were not appropriate. Two studies were judged with an unclear risk of bias, as no information was provided.
- Allocation concealment (selection bias): Three studies had a low risk of bias in this domain, as the allocation methods used were appropriate, and two studies were assessed with a high risk of bias because allocation was not concealed. In four studies, there was no information about allocation concealment procedures, resulting in an unclear risk of bias.
- Blinding of outcome assessment (detection bias): In four studies, the outcome assessor was unaware of the participants’ assigned interventions, so the risk of bias was low. Two studies were judged with a high risk, as the same therapists provided both treatments and assessments. In three studies, the risk was unclear due to lack of information.
- Incomplete outcome data (attrition bias): Eight studies were assessed with a low risk of bias for this domain, as no missing data were found. Only one study had an unclear risk of bias because the number of dropouts was not reported and potential missing data were not provided.
- Selective reporting (reporting bias): In six studies, the risk of bias was low, whereas in the remaining three studies the risk was unclear, as the study protocols were not available.

### Effects of intervention

#### Comparison 1. Cognitive domain, global. Outcome: Mini Mental State Exam. Telerehabilitation versus face-to-face treatment

**Figure 3:**
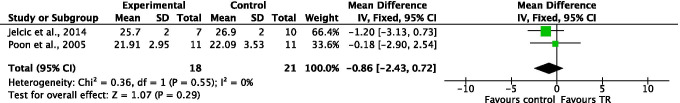
Comparison 1. Cognitive domain, global (Mini Mental State Exam): telerehabilitation vs. conventional face-to-face treatment. SD: standard deviation; 95% CI: 95% confidence interval

Two studies, with 39 participants overall, were analyzed for global cognitive domain, through analysis of the results from Mini Mental State Exam (MMSE). The analysis was performed using mean difference (MD) with fixed effect model and confidence interval (CI) of 95%. The meta-analysis did not show significant difference between the two treatment modalities (MD = −0.86; 95% CI −2.43, 0.72, *I*^2^ = 0%) (Figure 3).

#### Comparison 2. Learning and memory. Outcome: improvement in learning and memory abilities. Telerehabilitation versus conventional face-to-face treatment

**Figure 4:**
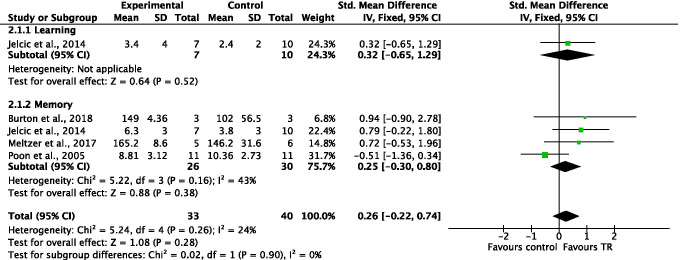
Comparison 2. Learning and memory (improvement in learning and memory abilities): telerehabilitation vs. conventional face-to-face treatment. SD: standard deviation; 95% CI: 95% confidence interval

A total of four studies, with an overall number of 73 participants, were analyzed, in order to evaluate improvement in learning and memory abilities. A subgroup analysis was performed, with regard to the analyzed ability (i.e., one study for learning ability, four studies for memory domain). The analyses were performed using standardized mean difference (SMD) with fixed effect model, since all the included studies used different outcome measures for the same outcome. No significant differences were found between TR and conventional face-to-face treatment for learning abilities (SMD = 0.32, 95% CI −0.65, 1.29, *I*^2^ = N/A), for memory domain (SMD = 0.25, 95% CI −0.30, 0.80, *I*^2^ = 43%), or in total comparison (SMD = 0.26, 95% CI −0.22, 0.74, *I*^2^ = 24%) (Figure 4).

#### Comparison 3. Language. Outcome: fluency. Telerehabilitation versus conventional face-to-face treatment

**Figure 5:**
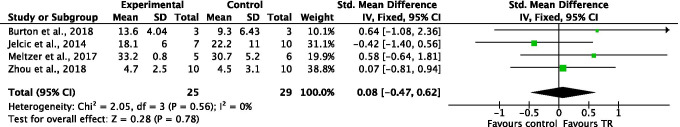
Comparison 3. Language (fluency): telerehabilitation vs. conventional face-to-face treatment. SD: standard deviation; 95% CI: 95% confidence interval

Four studies, comprising 54 participants, were included in the analysis of language ability, through the assessment of verbal fluency. Also in this case, analysis was performed using SMD with fixed effect model, and there were no significant differences between TR and face-to-face treatment (SMD = 0.08, 95% CI −0.47, 0.62, *I*^2^ = 0%) (Figure 5).

#### Comparison 4. Executive functions. Outcomes: problem-solving, central processing speed, working memory. Telerehabilitation versus conventional face-to-face treatment

**Figure 6:**
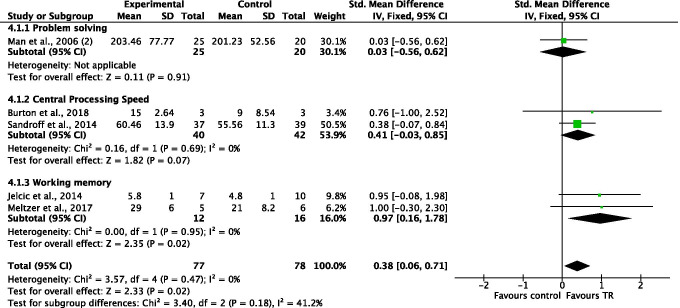
Comparison 4. Executive functions (executive functions): telerehabilitation vs. conventional face-to-face treatment. SD: standard deviation; 95% CI: 95% confidence interval

For the executive function domain, three subgroups were created, based on different mental skills evaluated (i.e., problem-solving, central processing speed, working memory). The overall number of participants analyzed from the five included studies was 155. Meta-analyses showed no significant differences between the two modalities for both problem-solving skill (SMD = 0.03, 95% CI −0.56, 0.62, *I*^2^ = N/A) and central processing speed (SMD = 0.41, 95% CI −0.03, 0.85, *I*^2^ = 0%). However, a statistically significant difference in favor of TR was found with regard to working memory (SMD = 0.97, 95% CI 0.16, 1.78, *I*^2^ = 0%) and in total comparison (SMD = 0.38, 95% CI 0.06, 0.71, *I*^2^ = 0%) (Figure 6).

## Discussion

This systematic review aimed to analyze and synthesize the evidence of the efficacy of cognitive TR interventions in patients with neurological diseases, compared to conventional face-to-face rehabilitation. We evaluated improvements in global cognitive domain, in learning and memory abilities, in language functions, and in executive functions. In all the included studies, improvement in performance of the TR groups was comparable to that achieved through face-to-face intervention. Significant differences between those two modalities of providing treatments were observed for working memory and total executive function comparison, in favor of TR.

However, some considerations are needed in relation to possible factors that could have influenced the results. In neurological patients, especially in older ones, hearing and vision impairments may interfere with some aspects of telecommunication logistic, such as visual quality or clarity. These factors could have influenced participants’ performance in TR groups by creating a condition where those patients were disadvantaged in the learning process, with consequences for the learning and memory abilities involved in the rehabilitation process [12]. These results have to be considered together with the benefits that TR has on social and economic aspects. Indeed, there are numerous potential advantages of using TR, such as reduced travel time, cost reductions, and access to services otherwise unavailable [19], especially for underserved population and for neurological patients with motor impairments, which negatively influence their mobility and their capacity to reach rehabilitation centers. In this context, TR becomes fundamental, and the results we found with this review can help clinicians to orient themselves to the best application of TR for the treatment of cognitive impairments in neurological patients.

The value of the conducted analysis is particularly apparent in the era of the COVID-19 pandemic, as it was found that TR training seems to be non-inferior to conventional face-to-face treatment, and similar goals can be achieved irrespective of whether treatment is provided via videoconferencing or through in-person contact. In the current situation, this is of great importance. It is no longer just a question of the problem of getting to therapy concerning certain groups of patients, but a complete lack of such possibility in case of a lockdown. Previous concerns regarding weaknesses of TR included, for instance, the limitation of social contacts resulting from the fact that the patient does not leave home even for therapy sessions. At present, TR may actually be seen as one of the ways of maintaining social contacts, which further emphasizes its advantages, when conducted via videoconference with a real therapist. However, due to the nature of TR application, it may still be an important way to break the monotony of daily routine, especially in front of the research, which demonstrates that the effects they bring in individual cognitive function domains are comparable to those achieved through face-to-face contact interventions.

### Study limitations

This study has some limitations that need to be addressed. Firstly, the included studies involved small sample sizes, highlighting the need to develop trials with larger population size. Secondly, although our secondary outcomes were related to the assessment of quality of life, patients’ satisfaction, feasibility, and cost-effectiveness of TR, only one study [5] reported results on patients’ quality of life, whereas no studies assessed satisfaction levels, feasibility, and cost-effectiveness of TR. Thirdly, insufficient data were reported in three studies. Two of them were finally excluded from meta-analysis, as only one author provided adequate data [16].

## Conclusion

TR is an emerging modality for the delivery of cognitive, motor, or linguistic treatment, especially in this pandemic moment, where the need to ensure the continuity of care is stressed. Indeed, TR has the potential to facilitate access to services and to give continuity to treatment, without decreasing its intensity and frequency after discharge from the hospital. The results of this study can sustain the efficacy of TR and its application for the treatment of neurological patients, especially when treated for executive functions’ impairments. Conversely, there is insufficient evidence across the included studies to reach a conclusion on the superiority of TR for global cognitive domain, language functions, and learning and memory abilities. However, this systematic review highlights the need for further research into TR use for cognitive impaired patients, in order to develop more powered trials and to improve the methodological quality of the evidence.

## Supplementary Information

Below is the link to the electronic supplementary material.

Supplementary file1 (DOCX 12 KB)

Supplementary file2 (DOCX 77 KB)

## Data Availability

Not applicable
