# Supplementary material for: Cognitive telerehabilitation in neurological patients: systematic review and meta-analysis
Source: Neurol Sci. 2021 Nov 25;43(2):847–62. doi: 10.1007/s10072-021-05770-6 (PMC8613517; doi:10.1007/s10072-021-05770-6)
Supplement: Supplementary file 1 — Supplementary file1 (DOCX 12 KB) [file 10072_2021_5770_MOESM1_ESM.docx]

**APPENDIX A – SEARCH STRATEGY**

**PUBMED**

1. ((((“Cognitive disorders” OR “Cognitive impairment” OR “Cognitive function” OR “Cognitive dysfunction” OR “Cognitive deficit” OR "Neurocognitive Disorders"[Mesh] OR "cognition disorders"[MeSH] OR "cognitive dysfunction"[MeSH] OR "Cognition"[Mesh]))) AND ((Telemedicine OR Telehealth OR Telerehabilitation OR teletherapy OR telepractice OR Teletreatment* OR Videoconferenc* OR “Distance education” OR Telemedicine [Mesh] OR Teleconferenc* OR “Virtual conference” OR “Cognitive exercises” OR “Cognitive training” OR “Cognitive rehabilitation” OR “Cognitive Therapies”))) AND (("Neuropsychological Tests"[Mesh] OR "Mental Status and Dementia Tests"[Mesh] OR “Mini Mental State Examination” OR “Montreal Cognitive Assessment” OR “Mini Mental Status Examination” OR “Assessment of cognitive functions” OR “Improvement in cognitive domains” OR “neuropsychological test*” OR “mental status test*”)) Sort by: Author Filters: Randomized Controlled Trial

**SCOPUS**

( TITLE-ABS- KEY ( “Cognitive disorders” OR “Cognitive impairment” OR “Cognitive function” OR “Cognitive dysfunction” OR “Cognitive deficit” OR "Neurocognitive Disorders" OR "cognition disorders" OR "cognitive dysfunction" OR "Cognition" ) AND DOCTYPE ( ar )) AND ( TITLE-ABS-KEY ( Telemedicine OR Telehealth OR Telerehabilitation OR teletherapy OR telepractice OR Teletreatment* OR Videoconferenc* OR “Distance education” OR Teleconferenc* OR “Virtual conference” OR “Cognitive exercises” OR “Cognitive training” OR “Cognitive rehabilitation” OR “Cognitive Therapies”) AND DOCTYPE ( ar )) AND ( TITLE-ABS-KEY ( Neuropsychological Tests" OR "Mental Status and Dementia Tests" OR “Mini Mental State Examination” OR “Montreal Cognitive Assessment” OR “Mini Mental Status Examination” OR “Assessment of cognitive functions” OR “Improvement in cognitive domains” OR “neuropsychological test*” OR “mental status test*”) AND DOCTYPE ( ar ))

**WEB OF SCIENCE**

#1 WC=(Telecommunications)

#2 WC=(Psychology, Multidisciplinary)

#3 WC=(Psychology)

#4 WC=(Rehabilitation)

#5 (#1 OR #2 OR #3 OR #4)

#6 TS=(“Cognitive disorders” OR “Cognitive impairment” OR “Cognitive function” OR “Cognitive dysfunction” OR “Cognitive deficit” OR "Neurocognitive Disorders" OR "cognition disorders" OR "cognitive dysfunction" OR "Cognition")

#7 TS=(Telemedicine OR Telehealth OR Telerehabilitation OR teletherapy OR telepractice OR Teletreatment* OR Videoconferenc* OR “Distance education” OR Teleconferenc* OR “Virtual conference” OR “Cognitive exercises” OR “Cognitive training” OR “Cognitive rehabilitation” OR “Cognitive Therapies”)

#8 TS=(Neuropsychological Tests" OR "Mental Status and Dementia Tests" OR “Mini Mental State Examination” OR “Montreal Cognitive Assessment” OR “Mini Mental Status Examination” OR “Assessment of cognitive functions” OR “Improvement in cognitive domains” OR “neuropsychological test*” OR “mental status test*”)

#9 (#5 AND #6 AND #7 AND #8)

**COCHRANE LIBRARY**

#1MeSH descriptor: [Neurocognitive Disorders] explode all trees

#2MeSH descriptor: [cognition disorders] explode all trees

#3MeSH descriptor: [cognitive dysfunction] explode all trees

#4MeSH descriptor: [Cognition] explode all trees

#5 (“Cognitive disorders” OR “Cognitive impairment” OR “Cognitive function” OR “Cognitive dysfunction” OR “Cognitive deficit”)

#6 (#1 OR #2 OR #3 OR #4 OR #5)

#7MeSH descriptor: [Telemedicine] explode all trees

#8 (Telemedicine OR Telehealth OR Telerehabilitation OR Videoconferenc* OR Teletherapy OR “Distance Education” OR Teleconferenc* OR Telepractice OR Teletreatment* OR “Virtual conference” OR “Cognitive exercises” OR “Cognitive training” OR “Cognitive rehabilitation” OR “Cognitive Therapies”)

#9 (#7 OR #8)

#10MeSH descriptor: [Neuropsychological Tests] explode all trees

#11MeSH descriptor: [Mental Status and Dementia Tests] explode all trees

#12 (“Mini Mental State Examination” OR “Montreal Cognitive Assessment” OR “Mini Mental Status Examination” OR “Assessment of cognitive functions” OR “Improvement in cognitive domains” OR “neuropsychological test*” OR “mental status test*”)

#13 (#10 OR #11 OR #12)

#14 (#6 AND #9 AND #13)

Tot. 809 trials

#15 ("randomized controlled trial"):ti,ab,kw

#16 (#14 AND #15)

**EMBASE**

1. ‘Neurocognitive Disorders’/de OR ‘cognition disorders’/de OR ‘cognitive dysfunction’/de OR ‘Cognition’/de

2. ‘Cognitive disorders’ OR ‘Cognitive impairment’ OR ‘Cognitive function’ OR ‘Cognitive dysfunction’ OR ‘Cognitive deficit’

3. (#1 OR #2)

4. ‘Telemedicine’/de

5. (‘Telemedicine’ OR ‘Telehealth’ OR ‘Telerehabilitation’ OR ‘Videoconferenc*’ OR ‘Teletherapy’ OR ‘Distance Education’ OR ‘Teleconferenc*’ OR ‘Telepractice’ OR ‘Teletreatment*’ OR ‘Virtual conference’ OR ‘Cognitive exercises’ OR ‘Cognitive training’ OR ‘Cognitive rehabilitation’ OR ‘Cognitive Therapies’)

6. (#4 OR #5)

7. ‘Neuropsychological Tests’/de OR ‘Mental Status and Dementia Tests’/de

8. (‘Mini Mental State Examination’ OR ‘Montreal Cognitive Assessment’ OR ‘Mini Mental Status Examination’ OR ‘Assessment of cognitive functions’ OR ‘Improvement in cognitive domains’ OR ‘neuropsychological test*’ OR ‘mental status test*’)

9. (#7 OR #8)

10. (#3 AND #6 AND #9)
