# Supplementary material for: Cognitive telerehabilitation in neurological patients: systematic review and meta-analysis
Source: Neurol Sci. 2021 Nov 25;43(2):847–62. doi: 10.1007/s10072-021-05770-6 (PMC8613517; doi:10.1007/s10072-021-05770-6)
Supplement: Supplementary file 2 — Supplementary file2 (DOCX 77 KB) [file 10072_2021_5770_MOESM2_ESM.docx]

**APPENDIX B**. Risk of bias assessment tables

**Burton et al., 2018**

| **Bias** | **Authors' judgement** | **Support for judgement** |
| --- | --- | --- |
| Random sequence generation (selection bias) |  | Random number generator |
| Allocation concealment (selection bias) |  | Sealed envelopes |
| Blinding of outcome assessment (detection bias) |  | No information about blinding outcome assessor |
| Incomplete outcome data (attrition bias) |  | Two participant from intervention group were lost during the study. Reasons for losses were disclosed. Losses unlikely to affect final results |
| Selective reporting (reporting bias) |  | All prespecified outcomes reported |

**Charvet et al., 2017**

| **Bias** | **Authors' judgement** | **Support for judgement** |
| --- | --- | --- |
| Random sequence generation (selection bias) |  | Stratified, permuted, block randomization |
| Allocation concealment (selection bias) |  | The study technician that assigned a participant's condition was not involved in the collection of data at baseline or study end visits |
| Blinding of outcome assessment (detection bias) |  | Outcome assessor blinded |
| Incomplete outcome data (attrition bias) |  | No dropouts |
| Selective reporting (reporting bias) |  | All prespecified outcomes reported |

**Man et al., 2006 (2)**

| **Bias** | **Authors' judgement** | **Support for judgement** |
| --- | --- | --- |
| Random sequence generation (selection bias) |  | two-stage process of random sampling (drawn by lot from a list of services, then drawn by lot from patient/member lists) |
| Allocation concealment (selection bias) |  | No information |
| Blinding of outcome assessment (detection bias) |  | No information |
| Incomplete outcome data (attrition bias) |  | Low rate of drop-outs (6%), balanced between groups |
| Selective reporting (reporting bias) |  | study protocol not available |

**Poon et al, 2005**

| **Bias** | **Authors' judgement** | **Support for judgement** |
| --- | --- | --- |
| Random sequence generation (selection bias) |  | No information |
| Allocation concealment (selection bias) |  | No information |
| Blinding of outcome assessment (detection bias) |  | No Information |
| Incomplete outcome data (attrition bias) |  | All outcome data reported |
| Selective reporting (reporting bias) |  | All pre-specified outcomes reported |

**Sandroff et al., 2014**

| **Bias** | **Authors' judgement** | **Support for judgement** |
| --- | --- | --- |
| Random sequence generation (selection bias) |  | random numbers generator and allocation by a person who was uninvolved in testing and intervention delivery |
| Allocation concealment (selection bias) |  | random numbers generator and allocation by a person who was uninvolved in testing and intervention delivery |
| Blinding of outcome assessment (detection bias) |  | All testing was administered by laboratory personnel who were not blinded to condition |
| Incomplete outcome data (attrition bias) |  | Missing outcome data were low and balanced in numbers across intervention groups |
| Selective reporting (reporting bias) |  | All outcome data were reported |

**Jelcic et al., 2014**

| **Bias** | **Authors' judgement** | **Support for judgement** |
| --- | --- | --- |
| Random sequence generation (selection bias) |  | The unequal distribution among the three treatment groups was due to the preference of two patients, initially enrolled in the LSS-tele group, to not be involved with computer technology and who were shifted into the other two treatment arms. |
| Allocation concealment (selection bias) |  | The unequal distribution among the three treatment groups was due to the preference of two patients, initially enrolled in the LSS-tele group, to not be involved with computer technology and who were shifted into the other two treatment arms. |
| Blinding of outcome assessment (detection bias) |  | All the assessments were carried out by an experienced neuropsychologist (SP), blinded to the treatment group to which each patient was allocated. |
| Incomplete outcome data (attrition bias) |  | no missing outcome data |
| Selective reporting (reporting bias) |  | all of the study’s pre-specified (primary and secondary) outcomes that are of interest in the review have been reported |

**

**

**Meltzer et al., 2017**

| **Bias** | **Authors' judgement** | **Support for judgement** |
| --- | --- | --- |
| Random sequence generation (selection bias) |  | Participants within each diagnosis category were randomized to either IP or TR.The randomized design demanded that some participants be assigned to the TR group regardless of what technological resources they had at home, as using that information to influence group assignment could have introduced a systematic confound in the results. |
| Allocation concealment (selection bias) |  | The randomized design demanded that some participants be assigned to the TR group regardless of what technological resources they had at home, as using that information to influence group assignment could have introduced a systematic confound in the results. |
| Blinding of outcome assessment (detection bias) |  | Initial and final assessments were carried out in person by an experienced SLP practitioner not involved in the treatment administration. |
| Incomplete outcome data (attrition bias) |  | No missing outcome data (controllare paragrafo comparison and non-inferiority testing : effect size) |
| Selective reporting (reporting bias) |  | include all expected outcomes |

### Torrisi et al., 2019

| **Bias** | **Authors' judgement** | **Support for judgement** |
| --- | --- | --- |
| Random sequence generation (selection bias) |  | Patients were randomized in order of recruitment |
| Allocation concealment (selection bias) |  | Concealment procedure not described |
| Blinding of outcome assessment (detection bias) |  | The trial was designed as a pilot, prospective, assessor-blinded, parallel-group study |
| Incomplete outcome data (attrition bias) |  | Number of drop out not reported, potential missing data not provided |
| Selective reporting (reporting bias) |  | Study protocol is not available, primary and secondary were not pre-specified |

**Zhou et al., 2018**

| **Bias** | **Authors' judgement** | **Support for judgement** |
| --- | --- | --- |
| Random sequence generation (selection bias) |  | No information |
| Allocation concealment (selection bias) |  | No information |
| Blinding of outcome assessment (detection bias) |  | Lack of blinding |
| Incomplete outcome data (attrition bias) |  | All participants included in the analysis |
| Selective reporting (reporting bias) |  | Study protocol is not available |
